# The Impact of Intensive Fish Farming on Pond Sediment Microbiome and Antibiotic Resistance Gene Composition

**DOI:** 10.3389/fvets.2021.673756

**Published:** 2021-05-25

**Authors:** Eglė Lastauskienė, Vaidotas Valskys, Jonita Stankevičiūtė, Virginija Kalcienė, Vilmantas Gėgžna, Justinas Kavoliūnas, Modestas Ružauskas, Julija Armalytė

**Affiliations:** ^1^Institute of Biosciences, Life Sciences Center, Vilnius University, Vilnius, Lithuania; ^2^Institute of Biochemistry, Life Sciences Center, Vilnius University, Vilnius, Lithuania; ^3^Institute of Microbiology and Virology, Lithuanian University of Health Sciences, Kaunas, Lithuania

**Keywords:** antibiotic resistance genes, fish farming, heavy metals, sediment microbiomes, sediment toxicity

## Abstract

Aquaculture is a fast-growing animal food sector, and freshwater fish farming is particularly common in Central and Eastern Europe. As the biodiversity of fishery ponds is changed toward fulfilling the industrial needs, precautions should be taken to keep the system sustainable and protect the adjacent environment from possible damage. Due to risk of infectious diseases, antibiotics are used in aquaculture production systems. The constant exposure to antimicrobials can contribute to the rise of antibiotic resistance in aquaculture products and the adjacent ecosystems, with possibility of dissemination to the wider environment as well as between animals and humans. Even though previous studies have found antibiotic resistance genes in the sediments and water of farming ponds, the tendency and direction of spreading is not clear yet. The objective of this project was to evaluate the influence of intensive fish farming on the condition of water bodies used for the aquaculture and the environment, concentrating on the impact of the aquaculture on the surrounding water ecosystems as well as the possibility of transferring the pollutants and antibiotic resistance genes to both environment and the human hosts. Combined measurement of antibiotic and heavy metal contamination, toxicity assessment, microorganism diversity, and the detection of common antibiotic resistance genes was performed in the sediments of one fishery farm ponds as well as sampling points upstream and downstream. All the tested sediment samples did not show significantly elevated heavy metal concentrations and no substantial veterinary antibiotic pollution. From the antibiotic resistance genes tested, the presence of aminoglycoside and β-lactam resistance determinants as well as the presence of integrons could be of concern for the possibility of transfer to humans. However, despite the lack of heavy metal and antibiotic pollution, the sediments showed toxicity, the cause of which should be explored more.

## Introduction

According to the report of Food Agriculture Organization of the United Nations 2020 aquaculture is one of the most important food sectors which, increased by annual 3.1% during 1961–2017 and exceeded annual world population growth (1.6%) almost double for the same period. Fish ponds are rich in dissolved organic materials due to the intensive feeding and fecal waste. Ponds continuously accumulate sediments after the formation of their basins with the influence of water regime (filling and discharging). These sediments are formed from biological remains originating in the ponds and its catchment area as well as soil particles and other non-biological materials that were transported to the pond. The most common type of sediments that is found in ponds is organogenic sediments (1). The intensity and combination of these processes are very variable depending on the different geological and geomorphological settings, hydrological regimes, and atmospheric conditions, as well as human activities (2, 3). The composition of sediments of aquaculture ponds could also be influenced by changes in the biodiversity, as the aquaculture is directed toward fulfilling the industrial needs. The monoculture of highly-productive industrial aquatic organisms is introduced and sustained by intensive rearing system, differing greatly from wild aquatic ecosystems.

Another factor that should be considered in the sediments of the fishery ponds is the presence of heavy metals. Heavy metals which enter aquatic environment typically bond with bottom sediments and, thus over time, can reach high concentrations. In these circumstances heavy metals can become a potential risk to human health through the food chain (4).

Heavy metal toxicity is of great ecological concern, due to their stability, bioaccumulation and non-biodegradability. The accumulation of the heavy metals can lead to the changes in microbial community composition and activation and accumulation of heavy metals resistance genes, that are often closely related to antibiotic resistance genes (5–9). It has been previously observed, that the co-selection of heavy metal and antibiotic resistance genes (ARGs) are happening in the environment (5), thus increasing the concern of the accumulation and spread of potentially hazardous ARGs from the environment to humans.

A more straightforward influence on accumulation and spread of ARGs in aquaculture is the use of antimicrobial substances for animal treatment. In the Eurozone, the use of veterinary drugs is regulated through EU Council Regulations (10, 11), which describe procedures for establishing maximum residue limits for veterinary medicinal products in foodstuffs of animal origin. In Lithuania, only two broad-spectrum antibiotics florfenicol and oxytetracycline are authorized for aquaculture use (http://vetlt1.vet.lt/vr/). Florfenicol is a structural analog of chloramphenicol similar to thiamphenicol, but is more active against some bacteria than chloramphenicol (12). Oxytetracycline is a tetracycline broad-spectrum antibiotic with bacteriostatic action, used to treat systemic bacterial infections of fish (13, 14). Among the 11 major aquaculture producing countries, about 73% applied oxytetracycline and florfenicol (15). The introduced antibiotics not consumed with the feed or excreted by the fed animals enter the water where they can persist or even concentrate in the sediments. The residual amounts of antibiotics in the environment have the potential to cause considerable impact on human health and ecosystems (16). However, these antibiotics are still not included in the (updated) Watch List of the Water Framework Directive (17). The antibiotic pollution problem deepens as farm animal manure can also be used to increase productivity of fishery ponds (18), thus introducing antibiotics used for animal treatment.

The analysis of sediments composition of long-running aquaculture farming is important for determination of the impact of anthropogenic activity and dynamics of pond ecosystems. The comparison of the sediments in the fishery ponds as well as upstream and downstream could show the impact of the intense aquaculture on the surrounding water ecosystems as well as the possibility of transferring the ARGs to the human hosts. We have chosen to analyze the sediments of Simnas fishery farm in Southern part of Lithuania, comparing them with samples in the inflow point located upstream from the fishery farm (chosen as an area untouched by antropogenic activity) and the outflowing river, carrying surplus water from the ponds. The aim of this study was to investigate the pollution of heavy metals and residual antibiotic in fishery ponds and the inflow and outflow points. Together with sediment composition analysis we explored the toxicity and determined the diversity and changes in microbiota composition as well as the presence of ARGs.

## Materials and Methods

### Sediment Sample Collection

**Figure 1:**
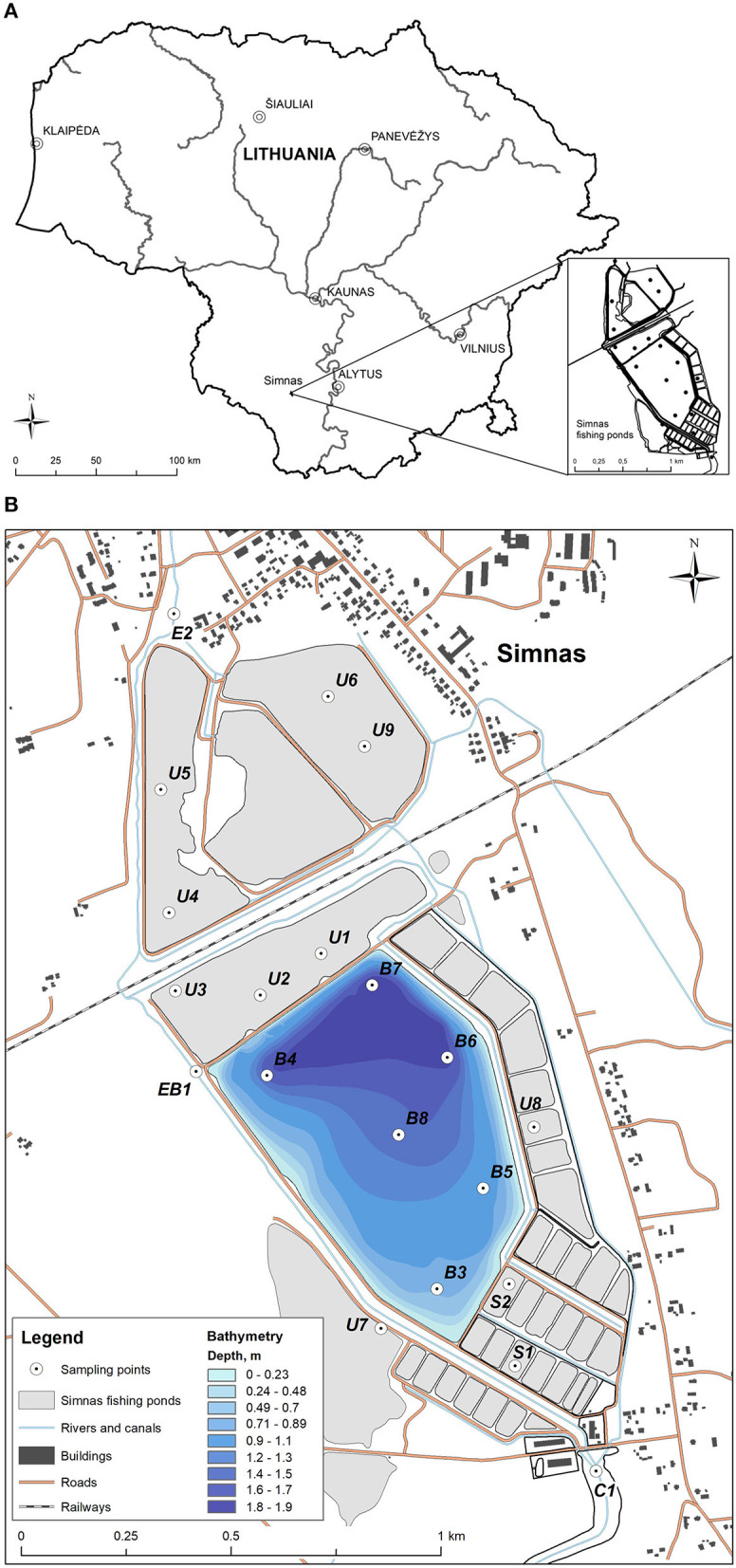
The sampling points in Simnas fishing ponds. **(A)** The location of Simnas fishery ponds in Lithuania, **(B)** The map of Simnas fishery ponds. Sample collection points are indicated and named in the figure, as well as the legend for the bathymetry measurements.

Sediment samples were collected during September 2019, from 20 sites in or near Simnas fishery ponds, located in Southern part of Lithuania. The sampling areas cover the inflow (Kalesnykai pond, C1) and outflow (Dovine river, E2) points of Simnas fishery ponds and 18 sample points directly in the fishing ponds (B1–B8 main fishery pond, BE1 exit from the main pond, U1–U8 unused ponds, S1–S2 nurseries). Sediment samples were collected from surface sediment layer using Kajak corer, registered and placed in plastic bags. The exact locations of the sampling points were recorded using the GNSS (Global Navigation Satellite System) device. In addition, altitudes of the main pond were registered and bathymetry was produced (Figure 1). ArcMap 10.8.1 software was used for mapping and geo-spatial analysis.

### Determination of Heavy Metal Concentration

Sediment samples were dried at 110°C to the constant mass, then the particles of the 125 μm size were separated and concentrations of HM (Heavy metals) were analyzed using X-ray fluorescence spectrometer NITON XL2 Analyzer (2009). The overall accuracy of chemical elements analyzed is between 10 and 20% for different chemical elements.

### Detection of Antibiotic Residues by HPLC-MS

Antibiotic residues were extracted from the sediment samples located in the main fishery pond and the exit areas, based on the method described previously (19). The sediment extracts were cleaned-up and concentrated using solid-phase extraction SAX cartridges (Merck, Germany) and HLB cartridges (Merck, Germany) in a tandem arrangement. The samples were eluted with 10 ml of methanol. Finally, the eluted samples were evaporated to dryness and dissolved in 1 ml of an aqueous 40% methanol solution (v/v). The resulting sediment extraction samples were analyzed using high-performance liquid chromatography-mass spectrometry (HPLC-MS) system (Shimadzu, Japan) equipped with a photodiode array (PDA) detector (Shimadzu, Japan) and mass spectrometer (LCMS-2020; Shimadzu, Japan) with an electrospray ionization (ESI) source. The chromatographic separation was conducted using a YMC Pack Pro column (3 × 150 mm; YMC, Japan) at 40°C and a mobile phase that consisted of 0.1% formic acid water solution (solvent A) and acetonitrile (solvent B) delivered in the 5–95% gradient elution mode. Mass scans were measured from m/z 50 up to m/z 1,200 at a 350°C interface temperature, 250°C desolvation line (DL) temperature, ±4,500 V interface voltage, and neutral DL/Qarray, using N_2_ as nebulizing and drying gas. Mass spectrometry data were acquired in both positive and negative ionization modes. The data were analyzed using LabSolutions liquid chromatography-mass spectrometry (LCMS) software.

### Sediment Toxicity Bioassay

The acute luminescent bacteria test was performed in compliance with ISO 11348-3:2007 using the *Aliivibrio fischeri* strain NRRL B-11177. The composition of bacterial culture growth medium and growth conditions were presented earlier (20). Biomass and suspension of marine *A*. *fischeri* for luminescence measurement was prepared as previously described (21). Sediment suspensions (solid-phase), aqueous elutriates and respective serial dilutions were prepared as described earlier (22). The exposure experiment started after addition of 20 μl bacteria suspension to each well containing 80 μl of prepared samples (sediment suspensions or elutriate supernatants at different concentrations), control (2% w/v NaCl) and reference chemical (3.5-dichlorphenol). The effect of elutriate supernatants and sediment suspensions was determined after 1 and 30 min, respectively, using microplate reader Tecan Infinite M200 (Tecan Group Ltd., Männedorf, Switzerland) at 20°C. Three independent measurements were conducted in duplicate. The level of luminescence inhibition in exposed groups was expressed as percentage relative to control according to formula: INH (%) = 100 − *BL*_*S*_/*BL*_*C*_ × 100; where *BL*_S_ bacterial luminescence after exposure to samples; and *BL*_C_ bacterial luminescence in control after respective incubation time. Median effective concentration (EC_50_ in mg dry weight/ml) of sediment suspensions was obtained using Regtox software (version EV7.0.5, Eric Vindimian, Paris, France). Since it was not possible to derive EC_50_ values for sediment elutriates it was replaced by the inhibition value after 1 min exposure to undiluted sediment elutriates corresponding to 75 mg dw sed./ml as suggested earlier (22). Solid-phase EC_50_ values were converted to toxic units (TU) values as follows: TU = 100/EC_50_. Samples were classified using Persoone et al. (23) classification system. Toxicity classes were determined according TU values estimated for solid-phase and percentage effect (PE) for sediments elutriates. No acute toxicity if PE < 20; slight acute toxicity if PE < 50; acute toxicity if 1 ≤ TU < 10; high acute toxicity if 10 ≤ TU < 100; very high acute toxicity if TU ≥ 100.

### DNA Extraction

Genomic DNA was isolated from sediment samples using the ZymoBIOMICS™ DNA Miniprep Kit (Zymo Research, USA) according to the manufacturer's recommendations. The concentration of extracted DNA was evaluated using a biophotometer (Eppendorf, Germany). Four DNA extractions were carried out for each sample. DNA was stored at −80 °C until further analysis. PCR inhibition was tested using primers Frrs/Rrrs (Supplementary Table 1).

### Microbiome Analysis

#### Sequencing

The composition of the bacterial community was determined by next-generation sequencing (NGS) by scanning the amplicons of the bacterial 16S rRNA gene. The V3–V4 16S rRNA regions were chosen for sequencing because they are capable to detect both bacterial and archaea taxons with high resolution (24, 25). NGS was performed by Novogene Bioinformatics Technology Co., Ltd. (Beijing, China) on Illumina paired-end platform to generate 250 base pairs (bp) length paired-end raw reads.

#### 16S rRNA Data Analysis

The reads were demultiplexed. Barcode and primer linker sequences were removed using “cutadapt” tool (26). The following steps were performed in QIIME2 (version 2020.11) (27). Data were denoised using read quality scores, low-quality part at the end of reads was trimmed (227 bp were left in forward and 224 bp in reverse reads), paired-end reads were merged and chimeras were removed using the pipeline that includes DADA2 algorithm (28). The result of DADA2 pipeline was amplicon sequence variants (ASV). Phylogenetic trees were created using MAFFT sequence alignment (29) and FastTree tree generation (30). Taxonomic, alpha and beta diversity analyses were based on ASV's. Taxonomic annotation was assigned by using pre-fitted Scikit-learn (31) based taxonomy classifier trained on full 16S gene (available at https://data.qiime2.org/2020.11/common/gg-13-8-99-nb-classifier.qza) based on Greengenes database (v13_8) at 99% threshold (32) *via* QIIME 2's “q2-feature-classifier” plugin (33). Core alpha and beta diversity metrics were generated with rarefaction depth equal to the lowest feature count of a single sample (34). Jaccard index was used as a measure of beta diversity.

### Antibiotic Resistance Gene Detection

Genes, commonly found in the clinically important bacteria and conferring resistance to the different classes of antibiotics used in the human and veterinary medicine, were included in the study. In addition, ARGs, previously found in the environmental samples were also screened. The presence of genes encoding antibiotic resistance determinants was assessed by PCR at the same conditions as described earlier (35). The genes tested and specific primers used are described in Supplementary Table 1. Together with ARGs detection, the presence of genes conferring resistance to heavy metals (As, Co, Cu, Pb, Cr) was also tested, the genes and primers are presented in Supplementary Table 1. PCR amplifying 16S rDNA fragment was used in parallel as amplification control.

## Results

### Heavy Metal Content Analysis

**Figure 2:**
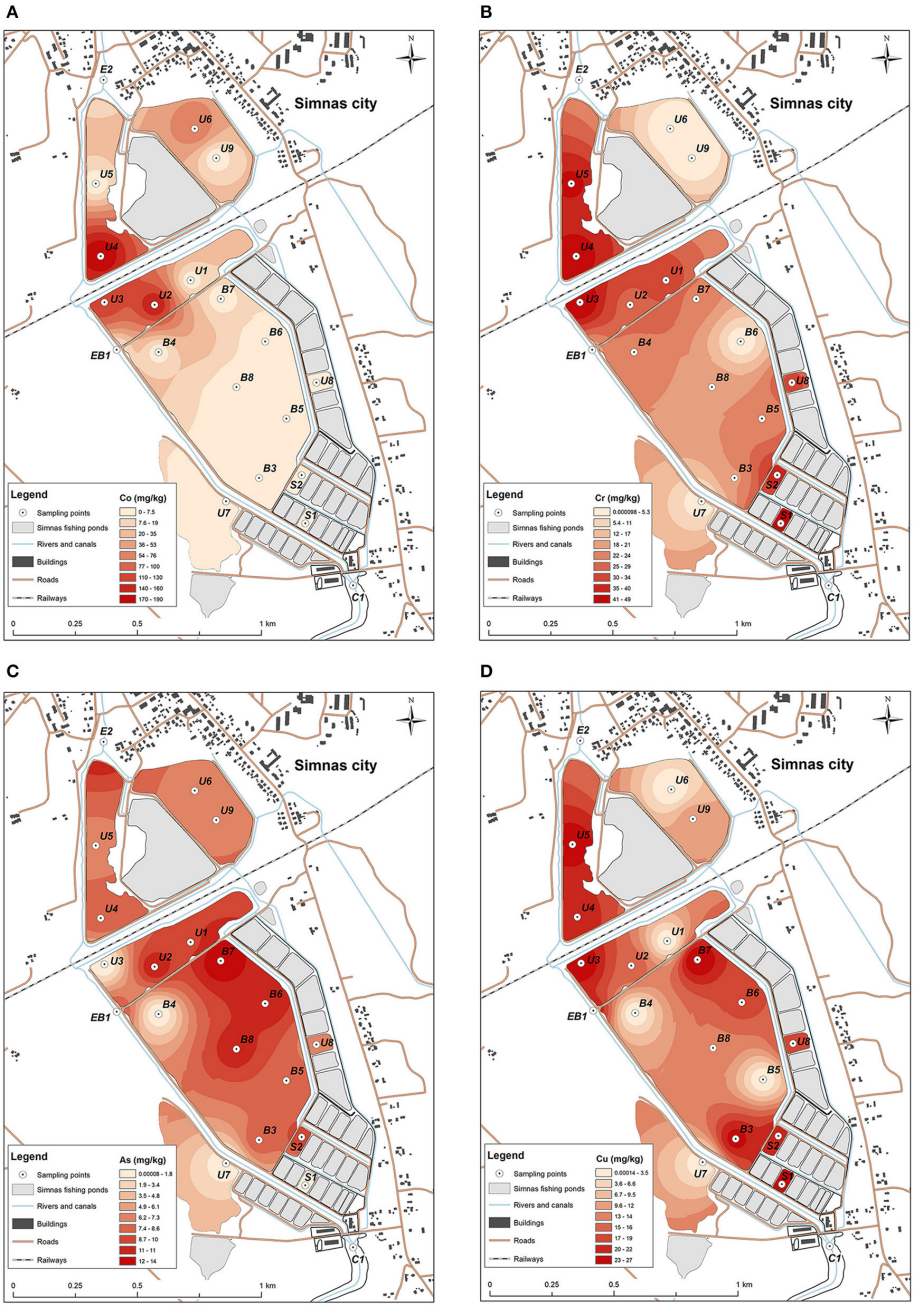
Distribution of heavy metals concentrations: **(A)** Co, with the highest concentration detected in U4, U3, and U2 sediment samples, **(B)** Cr, most abundant in small nursery ponds S1, S2, and U4, U5, U3, U8, **(C)** As, mostly detected in the sediments from the main pond at B8, B7, B6, and U2 sampling point from the unused pond, **(D)** Cu, prevalent in all the testes ponds, highest concentrations determined in U4, U5, U3, B3, B7, U8, S1 and S2 locations.

The concentrations of heavy metals that were detected in fishing ponds were similar to the geochemical composition of the bottom sediments of other Lithuanian lakes, with the exception of individual ponds located on both sides of the railway. The bottom sediments of these ponds were contaminated with Co and Cr which are common pollutants of railways (Figure 2). Co concentrations were up to 4 times (varies from 69.2 to 191.2 ± 23 mg/kg) higher than the maximum allowable concentrations (MAC) stated for lake bottom sediments. Concentrations of Cr did not exceed the MAC, but higher concentrations were also detected among both sides of the railway (varies from 18.8. to 53.9 ± 8.2 mg/kg).

An increase of As and Cu concentrations was also observed. Concentrations of these elements in the bottom sediments of the main fishing pond did not exceed the MAC, but a higher accumulation of these elements was observed in the northern part (concentrations of As varies from 6.1 to 13.7 ± 2.3 mg/kg, Cu—from 11.3 to 26.8 ± 6.4 mg/kg), due to the relief of the bottom of the pond (Figure 2), which descends from south to north. In this part, optimal conditions are formed for the sedimentation processes of bottom sediments (accumulation of sediments). In this way chemical elements are not removed or redistributed to other parts of the pond together with bottom sediments.

An analysis of the distribution of concentrations of chemical elements in different fisheries ponds showed that no influx of high concentrations of hazardous heavy metals could be observed during fish rearing activities. Only slight increase of As and Cu concentrations could be related with the activity of fishing ponds. The main sources of the pollution were the railway line crossing the territory of the fishery ponds and the nearby city of Simnas.

### Determination of the Antibiotic Residues in Sediments

Eight sediment samples (B3-B8, collected from the main fishery pond, and exit points BE1 and E2, located at the exit from the main fishery pond and the exit from the whole Simnas fishery farming, respectively) were analyzed for the presence of veterinary antibiotic residues by HPLC-MS. The characteristic molecular ions indicating oxytetracycline, florfenicol, and florfenicol amine (Supplementary Figures 1A–C) were not detected in any sediment sample tested, including sample no. B5 (Supplementary Figures 2, 3) which was chosen for representation. The limits of detection were 5.3, 9.2, and 15 ng/g in dry sediments for oxytetracycline, florfenicol, and florfenicol amine, respectively. Limits of detection were defined as the sample concentrations at a signal-to-noise ratio (S/N) of 3. Our findings indicate that the concentrations of oxytetracycline, florfenicol, and florfenicol amine in the sediment samples collected from Simnas fishery ponds were very low or below the detection limit.

### Sediment Toxicity

Solid-phase test results indicated that all tested sediment samples caused acute toxicity to *A*. *fischeri* (Table 1). Interestingly, the most toxic solid-phase of sediment sample was from Kalesnykai pond (C1), which was analyzed here as a clean entry point. The least toxic solid-phase of sample was collected from Exit site (E2). As it was expected due to complexity the solid-phase was more toxic than sediment elutriates. In case of undiluted aquatic sediment elutriates, the most part of analyzed samples did not inhibit the luminescence of *A. fischeri* bacteria, but enhanced light production (Table 2). Only one sample, which was collected in the main fishing pond (B7) caused slight acute toxicity.

**Table 1:** Solid-phase EC_50_ (mg sediment dry weight/ml), 30 min, determined using *A. fischeri* luminescence inhibition test.

**Sample No**.	**EC_**50**_, mg dry weight/ml, 30 min**	**Confidence interval, 95%**	**TU (toxic unit)**	**Classification according Persoone et al. (23)**
C1	18.71	12.21–25.77	5.35	acute toxicity
B3	38.05	37.90–38.30	2.63	acute toxicity
B4	21.94	21.81–22.01	4.56	acute toxicity
B5	52.53	50.29–54.40	1.90	acute toxicity
B6	45.11	38.52–49.78	2.22	acute toxicity
B7	25.63	23.40–28.72	3.90	acute toxicity
B8	51.60	47.50–54.33	1.94	acute toxicity
E2	62.94	62.94–62.94	1.59	acute toxicity

**Table 2:** Inhibition of *A. fischeri* luminescence caused by undiluted sediment elutriates corresponding to 75 mg sediment dry weight/ml, after 1 min incubation.

**Sample No**.	**Inhibition average, %**	**Standard deviation**	**Classification according Persoone et al. (23)**
C1	−66.15	0.18	No acute toxicity
B3	−7.70	3.48	No acute toxicity
B4	−3.52	10.50	No acute toxicity
B5	19.59	22.33	No acute toxicity
B6	15.80	3.09	No acute toxicity
B7	38.23	4.56	Slight acute toxicity
B8	16.60	5.37	No acute toxicity
E2	−54.94	33.33	No acute toxicity

### Microbiome Analysis

The number of species identified in one sample varied from 1,949 to 3,619 species for samples from ponds, 2,673 for samples at the entrances to the ponds (C1), as well as 3,619 and 3,450 for samples at the exit points (BE1, E2).

**Figure 3:**
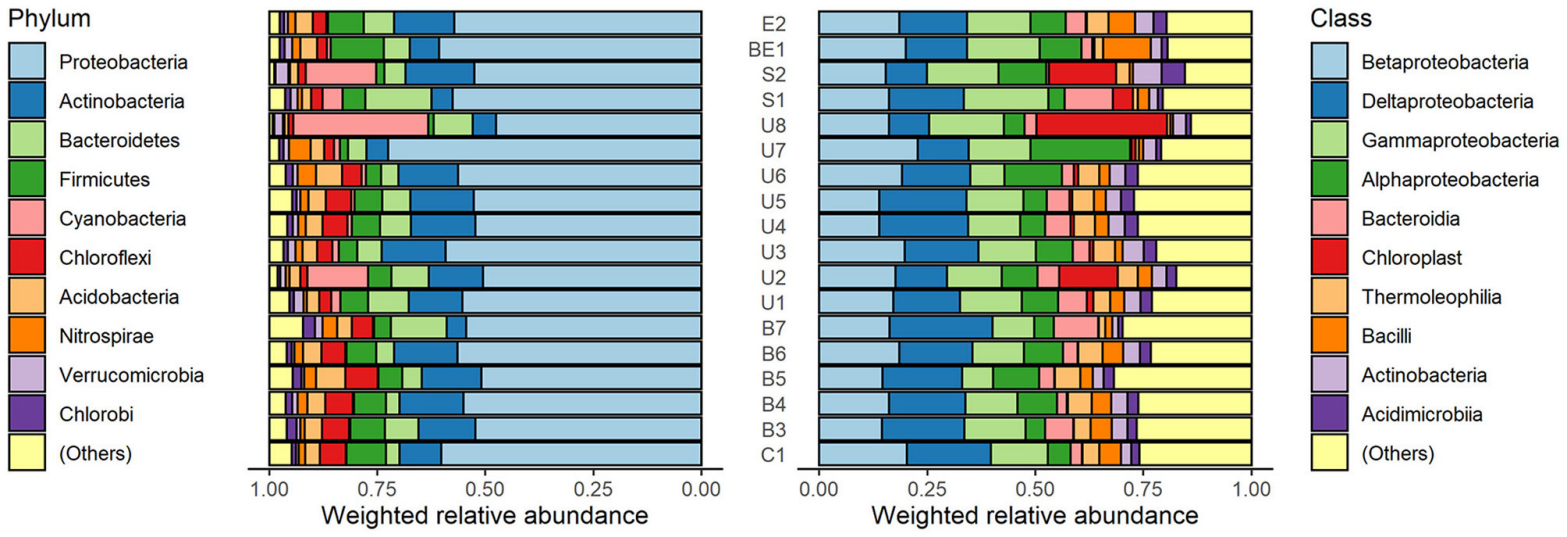
Weighted relative abundance of micro-organisms in phylum **(left)** and class **(right)** level in sediment specimens. Both phylum and class named “(Others)” represent relative abundance of the remaining phyla or classes besides the top 10 ones.

Sediment sample analysis showed that 10 phyla with highest average relative abundance of identified microorganisms were *Proteobacteria, Actinobacteria, Bacteroidetes, Firmicutes, Cyanobacteria, Chloroflexi, Acidobacteria, Nitrospirae, Verrucomicrobia*, and *Chlorobi* (Figure 3, left). *Proteobacteria* phylum microorganisms predominate in all samples (mean 55.8%, SD = 5.4%). The highest relative abundance of *Proteobacteria* was identified in the U7 sample (72.4%) and the lowest in the U8 sample (47.6%). *Actinobacteria* phylum microorganisms also make up a large part of the microorganism communities (mean 11.4%, SD = 4.1%). The highest number of these microorganisms was detected at the S2 point (15.6%) and the lowest at the B7 point (5.2%). *Bacteroidetes* abundance varies between 3.1 and 15.2%, *Firmicutes*−1.3–12.4%, *Chloroflexi*−1.1–7.6%, *Acidobacteria*−0.9–6.7% and form a significant proportion of bacterial communities. *Nitrospirae, Verrucomicrobia*, and *Chlorobi* were less frequently detected, with a <5% in relative abundance. It is important to note that *Cyanobacteria* are particularly characteristic of U2 (14.0%), U8 (31.2%), and S2 (16.3%) samples. At the remaining points, the cyanobacterial content did not exceed 5%.

The analysis of the relative abundance of the classes (Figure 3, right) revealed that dominant microorganisms in the samples belong to *alpha-proteobacteria* (3.7–23.0%), *beta-proteobacteria* (13.9–22.8%), *gamma-proteobacteria* (7.2–19.7%), and *delta-proteobacteria* (9.2–23.8%) classes. The abundance of the *Acidimicrobiia* class varies from 1.0% (B7) to 5.4% (S2). Small amount of *Thermoleophilia* (0.8–5.9%) was detected in all the samples, as well as *Bacilli* class microorganisms (0.6–11.1%).

**Figure 4:**
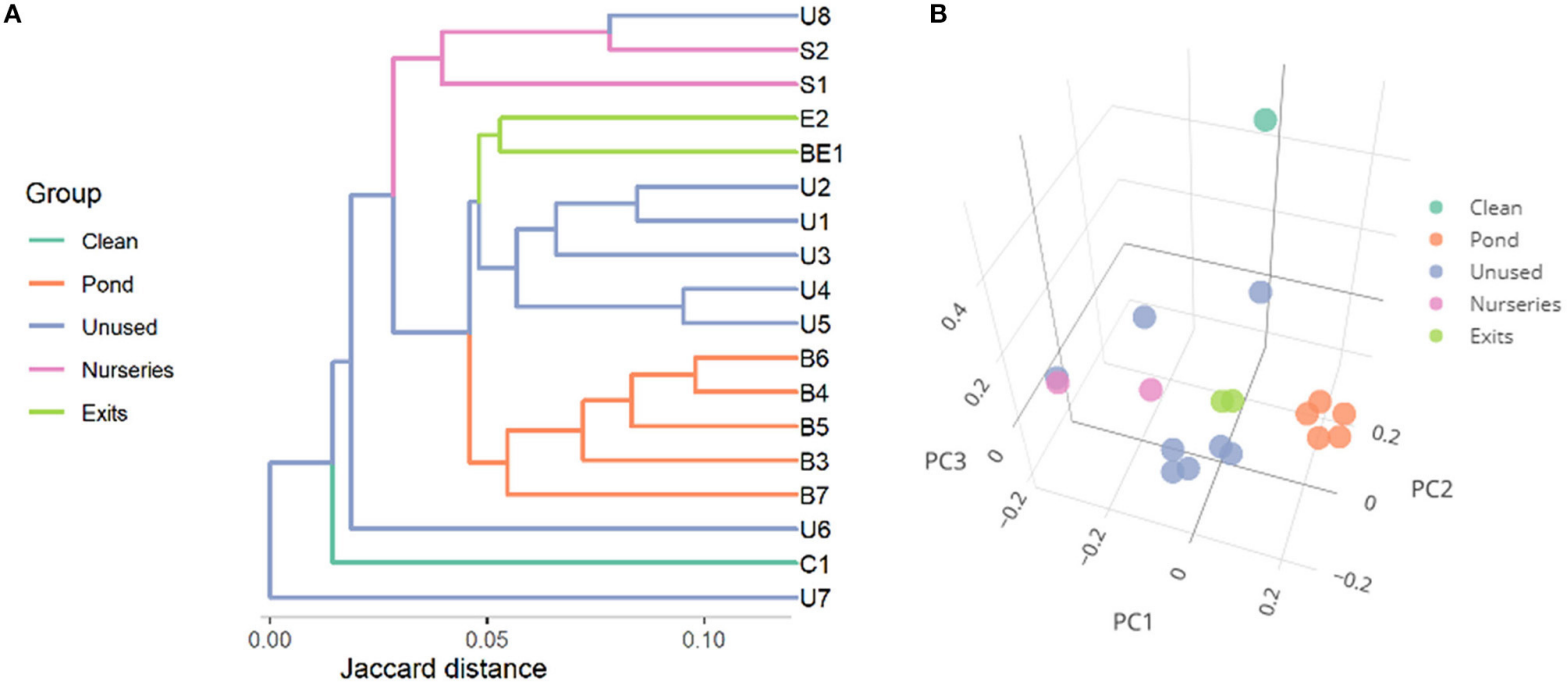
Microbial similarity (beta-diversity) of specimens expressed as Jaccard distances and represented as **(A)** a dendrogram and **(B)** principal coordinates plot. The first three principal coordinates (PC) explain, respectively, 10.8, 8.4, and 7.1% of variance.

The microbiome beta-diversity analysis results clearly indicated the differences between the microbiota composition of all pond sediments and entrance point, treated as a clean area (Figure 4). In the PC plot, the samples from the exits of the ponds were situated in the middle of the other sample points, and the points from the main pond (orange) formed a separate cluster. In this plot, the differences of the clean (taken upstream the ponds) and the remaining specimens were evident.

**Figure 5:**
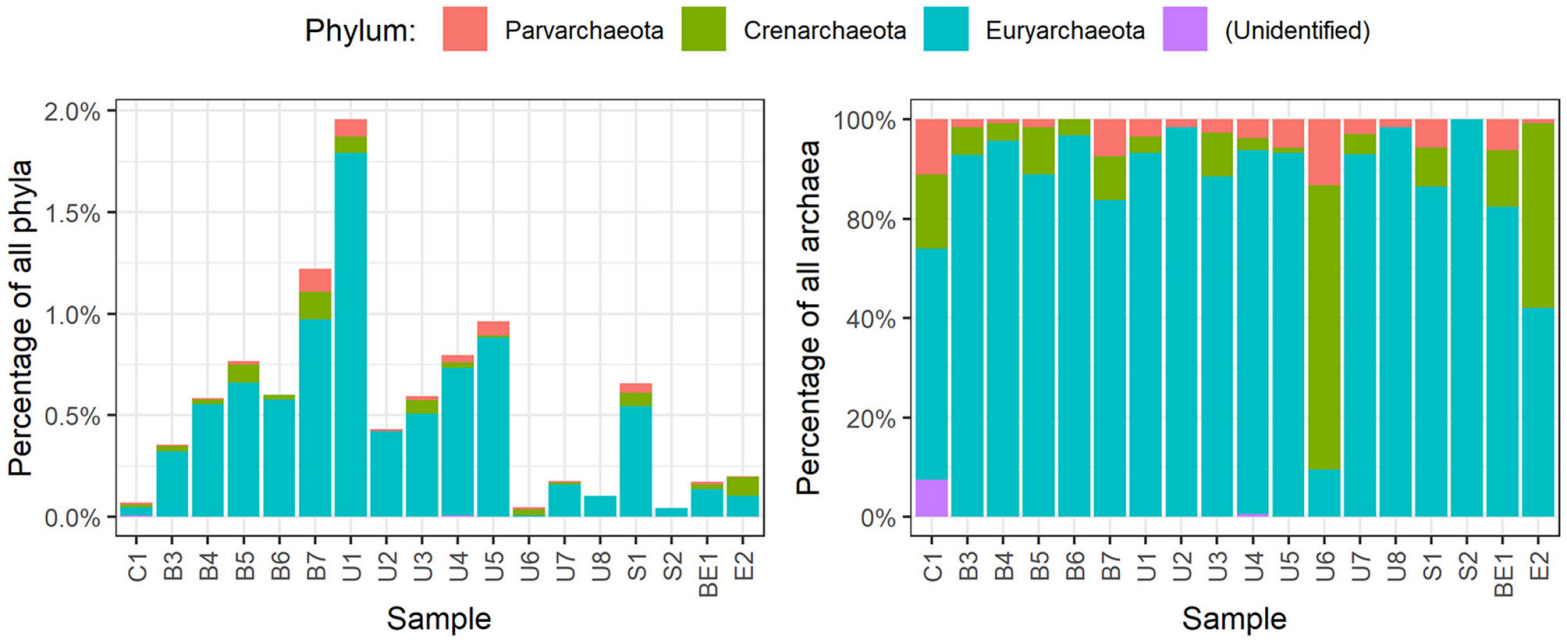
Distribution of archaea phyla in each specimen of sediments.

Members of *Archaea* domain were found in the sediments as well. Most of them belonged to phylum *Parvarchaeota, Crenarchaeota*, and *Euryarchaeota* (Figure 5). *Euryarchaeota* was the predominant phylum of *Archaea* found in all the specimens, with abundance varying from 0.1 to 1.1%. Highest abundance of *Archaea* (2.0%) was discovered in U1 sample (*Parvarchaeota* 0.1%, *Crenarchaeota* 0.1%, *Euryarchaeota* 1.8%).

### Antibiotic Resistance Gene Detection

**Figure 6:**
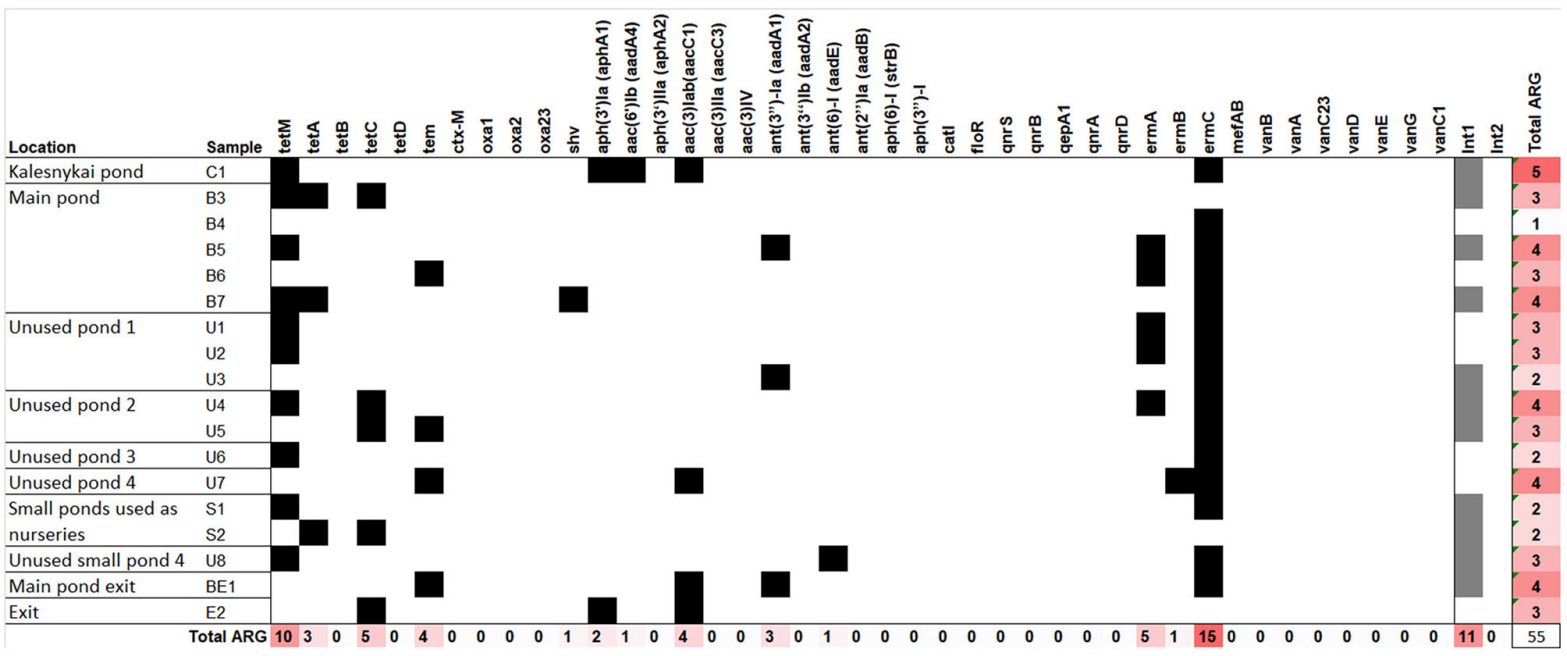
The antibiotic resistance genes detected in the total DNA of fishery pond sediments. Black squares denote the gene was detected. The same samples were tested for the presence of class I and II integrase genes, the presence of the genes is indicated by gray squares.

The ARG detection results are presented in Figure 6. Tetracyclin resistance genes were quite common in the sediment samples, the most common one being *tetM*, detected in more than a half of the samples. However, *tetM* was also found in entrance point sample (C1), indicating the spread of tetracyclin ARGs might not be related to fishery pond treatment. The screening for β-lactamase ARGs, revealed an extended spectrum β-lactamase (ESBL) *tem* gene, which was found in the samples both main pond and in the unused ponds, and one case of the *shv* ESBL gene. *shv* gene was detected in the sample B7 located in the deepest part of the pond where accumulation of sediments could occur (Figures 1, 6). Apart from ESBLs, the only genes of known clinical relevance were the ones coding for aminoglycoside modifying enzymes. *The aph(3*′*)-Ia, aac(6*′*)-Ib, aac(3)-Iab, ant(3*″*)-Ia and ant(6)-I*, genes, coding for a range of aminoglycoside resistance were detected, three of them in the entrance point of the fishery ponds C1. Macrolide resistance gene *ermC* was present in the majority of samples, while *ermA* and *ermB* were also detected. We have also tested for the most common heavy metal resistance genes (Supplementary Table 1), however, only one instance of *chrB* gene, coding for a regulator of Cr resistance operon, was detected in U3 sample (not shown), which represents one of the most Cr polluted areas in the ponds (Figure 2).

Since an important trait of both heavy metal resistance genes and ARGs is their ability to be transferred between the organism thus spreading the trait, we also tested the sediment samples for the presence of integrons. Only integrase genes belonging to class I were found in the sediment samples, spread evenly between main ponds and unused ponds and also in the C1 sample, indicating the presence of mobile elements in the fishery ponds as well as the adjacent areas.

## Discussion

In this study, we aimed to evaluate the condition of Simnas fishery ponds, that have been used for aquaculture since 1964. Only one main pond is currently used for fish farming, and several smaller ones are still used as nurseries, therefore we had an opportunity to see the differences in fishery pond sediments composition under intense use vs. unused for several years.

Most of the tested heavy metal concentration mostly did not exceed the MAC, only Co was detected up to 4-fold higher concentrations than MAC. The highest concentrations of Co and Cr was apparently due to railway line passing the fishery ponds territory. However, increased concentrations of Cr were also observed in small ponds used as nurseries (S1 and S2). Increased concentrations of Cu and As were mostly dependent on the descending relief of the ponds, concentrations increasing where the sediments collect. Concentrations of heavy metals in sediments of Simnas fishing ponds were similar to other already investigated lakes in Lithuania, situated in a moderate anthropogenic environment (36, 37) and were much higher than the background concentrations of natural and semi natural lakes (38). Though the concentration in the fishery pond samples did not exceed the MAC (39, 40), the observed heavy metal concentration could indicate the increase of over time is ongoing.

The presence of heavy metals in the environment is known to be connected with ARG co-selection (9). Even present in low levels, heavy metals and antibiotics could enhance the selection of bacteria carrying ARGs (41). Oxytetracycline accumulation in sediments has been reported at concentration levels of a few to hundreds of μg/kg in different water bodies (42, 43), reaching maximum concentration of hundreds μg/kg d.w. was found in sediments sampled near fish farms in Italy (44, 45). However florfenicol and its metabolite florfenicol amine can be detected in surface water but not in sediments of aquaculture systems (45). Our testing for the residues of two antibiotics, that are allowed for use in veterinary setting in Lithuania, also did not detect their presence in the main pond sediments.

Our results show only a minor accumulation of heavy metals and no substantial pollution with antibiotics, hopefully indicating no additional pressure on ARG co-selection. However, one case of Cr resistance gene was observed in the area of one of the highest Cr concentrations, indicating further increase of heavy metal concentration could push the microorganisms toward obtaining heavy metal resistance genes, which could be followed by co-selection of ARGs.

The sediment microbiome analysis revealed that *Proteobacteria* are the most abundant phylum found in all the sediment samples. Sediments from fishing ponds are commonly characterized by high concentrations of organic and inorganic substances. These substances settle to the bottom of ponds together with fish feces and uneaten feed and cause eutrophication of water bodies and depletion of oxygen. Our findings are in agreement with other authors indicating that *Proteobacteria* is the most frequent phylum found in water bodies and dominate between fishery ponds microorganisms (46, 47). *Proteobacteria* in aquaculture are known as organic-degrading microorganisms (47). Liu et al. found that *Proteobacteria* predominated in both water and sediment samples, regardless of the species farmed in the ponds and the aquaculture pattern. meanwhile, discovered that the use of different fish feeds also did not affect the dominance of *Proteobacteria*. The second type of bacteria in terms of the highest abundance is *Actinobacteria* (mean 11.4%, SD = 4.1%). These bacteria are also commonly found in water bodies (48). In addition, the abundance of *Actinobacteria* and *Firmicutes* is known to be positively correlated with sediment pH (46, 49). *Firmicutes*-type bacteria were characteristic of all studied groups of the samples, but most of them were detected in the BE1 sample (12.4%). Meanwhile, *Chloroflexi* microorganisms abundant in all samples play an important role in sediment carbon metabolism (50). An equally important process is the oxidation of fish-toxic nitrites to fewer toxic nitrates (51). *Nitrospirae*-type microorganisms are known to be able to catalyze these oxidation reactions and were found in all samples.

Analysis of the class structure in the samples revealed that the dominant type of *Proteobacteria* consists of microorganisms of the classes *Alfaproteobacteria, Betaproteobacteria, Gammaproteobacteria*, and *Deltaproteobacteria*. High abundance of *Gammaproteobacteria* is associated with an environment enriched with organic substances (46). *Deltaproteobacteria* also can be used as a bioindicator of organic compound contamination. *Betaproteobacteria*, are known as nitrifying bacteria, capable to oxidize potentially toxic ammonia to non-toxic nitrates. This process is particularly important in aquaculture ponds, where ammonia can reach concentrations harmful to fish (46). It is important to note that very few human pathogenic genera were identified in this study. Highest abundance of family *Listeriaceae* were found in E2 (2.29%) and B3 (2.50%) samples. In C1, B4, U6, U7, BE1 samples number didn't exceed 0.5% and *Listeriaceae* were not detected in the rest of the sampling points. *Listeria monocytogenes* is reported to be predominant in temperate aquaculture. *Listeria monocytogenes* can be found in lightly preserved or raw aquatic food products and become the cause of human disease (52). Other genera with the members exhibiting potential pathogenicity detected in our research are *Bacillus* and *Pseudomonas*, but their relative abundance in sediment samples is low.

The *Archaea* community analysis revealed the predominance of *Archaea* belonging to phylum *Euryarchaeota, Crenarchaeota*, and *Parvarchaeota*. The knowledge of uncultivated archaea, previously known as extreme environment microorganisms, revealed that they can be found in various environments from extreme to ordinary (53). They are an important part of the ecosystem capable of cycling of carbon, nitrogen, sulfur, and others playing the important role in the biogeochemical cycle of those elements (54). Many *Archaea* species are capable to fix carbon from inorganic sources and can affect the dynamics and balance of greenhouse effect related gases. Moreover, *Archaea* are the microorganisms capable to fulfill various metabolic strategies using organic and/or inorganic electron donors and acceptors (55).

ARG were not abundant in the fishery pond sediments, most of the ARGs found were the ones conferring resistance to tetracyclines, which could indicate the history of oxytetracycline use. However, the presence of tetracycline resistance genes has been previously observed also in pristine environments (56), therefore the connection with veterinary antibiotic use need to be analyzed further. A variety of aminoglycosides ARGs were detected, which confer resistance to various aminoglycosides, even the ones used in clinical setting (such as gentamicin, amikacin, tobramicin), which could be a reason for concern. However, the presence of three aminoglycoside genes in C1 sample, which was upstream from the fishery ponds, indicated the ARGs could be present as components of naturally inhabiting microorganisms. Our preliminary data indicate, aminoglycoside ARGs can be found also in the water bodies higher upstream from the fishery ponds, which would further confirm them being a part of the natural microbiota. From β-lactam ARGs *tem* was the most common, which has been observed elsewhere (57, 58), and one sediment sample (B7, located in the deepest part of main pond) also had *shv*. The presence of *shv* in fishery farming samples has also been reported previously (57). However, finding ESBL gene in the environment could always be considered a hazard due to the possibility of transferring it to humans by means of fish produce. Interestingly, *tetM, ermC* were also often found in the soil samples from Lithuanian farmland (59), indicating the spread of such ARGs in Lithuania or a natural habitat of the microorganism bearing them. The presence of integrons was also checked. More than a half of tested sediment samples contained integrons, as detected by the presence of integrase (class I) genes, indicated that the discovered ARGs could indeed be mobilized and transferred between the species, including human pathogens.

Even though the heavy metal concentrations did not exceed the MAC and residues of antibiotics were not detected, the toxicity of the samples has been observed. Inhibition of bacteria luminescence could be caused by mixtures of various components, which are at MAC or lower concentrations. Effects of elutriates and solid-phase reflect toxicity of water soluble compounds and whole sediments containing adsorbed chemicals, respectively. The determined toxicity of elutriates was lower than of solid-phase, such differences in toxicity have been observed earlier (22, 60). The solid-phase EC_50_ values (19–63 mg/ml) were similar to sediment toxicity results observed for contaminated river (22) and freshwater aquaculture (61). More than two orders of magnitude lower solide-phase EC_50_ values were determined for sediments of Atlantic coast of Spain (ranged of 0.051–20.23 mg/ml) and was highly affected by sulfide concentrations (60).

Altogether, no elevated heavy metal concentrations and no substantial veterinary antibiotic pollution was detected in Simnas fishery ponds. From the ARGs tested, the presence of aminoglycoside and β-lactam resistance determinants as well as the presence of integrons could be of concern. However, despite the lack of heavy metal and antibiotic pollution, the toxicity of the sediments and its cause should be explored more, as other compounds causing it could be affecting the health of fish population and consequently humans.

## Data Availability Statement

The datasets presented in this study can be found in online repositories. The names of the repository/repositories and accession number(s) can be found at: NCBI Bioproject PRJNA715198.

## Author Contributions

EL: sample preparation, total genomic DNA extraction, preparation of the manuscript, acquiring of funding, and interpretation of the metagenome analysis results. VV: sample collection and determination of the heavy metal concentrations. JS: antibiotic detection. VK: toxicity analysis. VG and JK: bioinformatic analysis of the metagenome data. MR: supervision and acquiring funding. JA: supervision of ARG detection, interpretation of antibiotic resistance results, funding acquisition, and preparation of manuscript. All authors contributed to the article and approved the submitted version.

## Conflict of Interest

The authors declare that the research was conducted in the absence of any commercial or financial relationships that could be construed as a potential conflict of interest.
