# Supplementary material for: The Impact of Intensive Fish Farming on Pond Sediment Microbiome and Antibiotic Resistance Gene Composition
Source: Front Vet Sci. 2021 May 25;8:673756. doi: 10.3389/fvets.2021.673756 (PMC8186532; doi:10.3389/fvets.2021.673756)
Supplement: Supplementary file 1 [file Data_Sheet_1.docx]

Supplementary Material

# Supplementary Figures and Tables

**Table S1.** Oligonucleotide primers used in the study.

| **Gene** | **Primer** | **Primer sequence 5‘-3‘** | **Amplicon size, bp** | **Reference** |
| --- | --- | --- | --- | --- |
| **rRNA** | Frrs | GATTAGATACCCTGGTAGTCC | 319 | (Armalytė et al., 2019) |
| 16S rRNA | Rrrs | GTTGCGGGACTTAACCCAAC |  |  |
|  |  |  |  |  |
| **β lactam ARGs** |  |  |  |  |
| *ctx-M* | FCTXU | ATG TGC AGY ACC AGT AAR GTK ATG GC | 593 | (Eckert et al., 2006) |
|  | RCTXU | TGG GTR AAR TAR GTS ACC AGA AYC AGC GG |  |  |
| *oxa1* | F1OXA1 | GAT ATC TCT ACT GTT GCA TCT C | 619 | (Fang et al., 2008) |
|  | R1OXA1 | AAT AAA CCC TTC AAA CCA TCC G |  |  |
| *oxa2* | FOXA2 | GCC AAA GGC ACG ATA GTT GT | 600 | (Fang et al., 2008) |
|  | ROXA2 | TCA TCC ATC CTG TTT GGC GT |  |  |
| *oxa23* | FOXA23 | TTA GCA CCT ATG GTA ATG CTC T | 526 | (Fang et al., 2008) |
|  | ROXA23 | TCC ACC CAA CCA GTC AAC CA |  |  |
| *shv* | F1SHV | AGG ATT GAC TGC CTT TTT GCG | 392 | (Fang et al., 2008) |
|  | R1SHV | ATT TGC TGA TTT CGC TCG GC |  |  |
|  |  |  |  |  |
| **Aminoglycosides ARGs** | | | | |
| *ant(6)-I (aadE)* | FANT-6 | AGCCGGAGGATATGGAATTAT | 463 | (Ramirez and Tolmasky, 2010) |
|  | RANT-6 | TTCATAGGAATCCATCCGGTA |  |  |
| *ant(3”)-Ia (aadA1)* | FANT-3 | CGC CGA AGT ATC GAC TCA AC | 559 | (Chen et al., 2004) |
|  | RANT-3 | GCG GGA CAA CGT AAG CAC TA |  |  |
| *ant(3‘‘)Ib (aadA2)* | FAAD2 | GCTCAATGACCTTATGAAGGC | 379 | (Šeputiene et al., 2006) |
|  | RAAD2 | GCGGGACAACGTAAGCACTA |  |  |
| *ant(2’’)Ia (aadB)* | FANTIa | GAGCGAAATCTGCCGCTCTG | 320 | (Vakulenko et al., 2003) |
|  | RANTIa | CTGTTACAACGGACTGGCC |  |  |
| *aph(6)-I (strB)* | FAPH -6 | ATC GTC AAG GGA TTG AAA CCT A | 510 | (Madsen et al., 2000) |
|  | RAPH -6 | GGA TCG TAG AAC ATA TTG GCG |  |  |
| *aph(3‘)Ia (aphA1)* | FAPHI | ATGGGCTCGCGATAATGTC | 634 | Sheryl et al., 2008 |
|  | RAPHI | CTCACCGAGGCAGTTCCAT |  |  |
| *aph(3‘)IIa (aphA2)* | FAPHII | GAACAAGATGGATTGCACGC | 680 | (Maynard et al., 2003) |
|  | RAPHII | GCTCTTCAGCAATATCACGG |  |  |
| *aph(3”)-I* | F1APH-3 | CTT GGT GAT AAC GGC AAT TCC | 547 | (Madsen et al., 2000) |
|  | R1APH-3 | CCA ATC GCA GAT AGA AGG CAA |  |  |
| *aac(3)Iab(aacC1)* | FAAC3I | AGCAGCAACGATGTTAACGCA | 470 | (Ramirez and Tolmasky, 2010) |
|  | RAAC3I | CTGCGGGATCGTCACCGTA |  |  |
|  |  |  |  |  |
|  |  |  |  |  |
| *aac(6’)-Ib(aadA4)* | FAAC6b | AGT ACA GCA TCG TGA CCA ACA | 500 | (Machado et al., 2006) |
|  | RAAC6b | ATG TAC ACG GCT GGA CCA TC |  |  |
| *aac(3)IIa (aacC3)* | FAAC3IIa | GGTT CGG CCT GCT GAA TCA | 442 | (Ramirez and Tolmasky, 2010) |
|  | RAAC3IIa | AA GCC CAC GAC ACC TTC TC |  |  |
| *aac(3)IV* | FAAC3IV | GATGGGCCACTTGGACTGAT | 462 | (Chen et al., 2005) |
|  | RAAC3IV | GCGCTCACAGCAGTGGTCAT |  |  |
| *aac(6‘)Ib (aadA4)* | FAAC6b | AGTACAGCATCGTGACCAACA | 500 | (Galimand et al., 1993) |
|  | RAAC6b | ATGTACACGGCTGGACCATC |  |  |
|  |  |  |  |  |
| **Tetracyclines ARGs** |  |  |  |  |
| *tetA* | FTETA | GCT ACA TCC TGC TTG CCT TC | 210 | (Ng et al., 2001) |
|  | RTETA | CAT AGA TCG CCG TGA AGA GG |  |  |
| *tetB* | FTETB | TTG GTT AGG GGC AAG TTT TG | 659 | (Ng et al., 2001) |
|  | RTETB | GTA ATG GGC CAA TAA CAC CG |  |  |
| *tetC* | FTETC | CTT GAG AGC CTT CAA CCC AG | 418 | (Ng et al., 2001) |
|  | RTETC | ATG GTC GTC ATC TAC CTG CC |  |  |
| *tetD* | FTETD | AAA CCA TTA CGG CAT TCT GC | 787 | (Ng et al., 2001) |
|  | RTETD | GAC CGG ATA CAC CAT CCA TC |  |  |
| *tetM* | FTETM | GTG GAC AAA GGT ACA ACG AG | 406 | (Ng et al., 2001) |
|  | RTETM | CGG TAA AGT TCG TCA CAC AC |  |  |
|  |  |  |  |  |
| **Macrolides ARGs** |  |  |  |  |
| *ermA* | FermA | GAAGCGGTAAACCCCTCTG | 216 | (Seputiene et al., 2012) |
|  | RermA | ACCCAAAGCTCGTTGCAGAT |  |  |
| *ermB* | FermB | ATTGGAACAGGTAAAGGGCAT | 447 | (Seputiene et al., 2012) |
|  | RermB | ATCTGGAACATCTGTGGTATG |  |  |
| *ermC* | FermC | GAAATCGGCTCAGGAAAAGG | 293 | (Seputiene et al., 2012) |
| *mefAB* | FmefAB | AGTATCATTAATCACTAGTGCC | 347 | (Seputiene et al., 2012) |
|  | RmefAB | GTTCTTCTGGTACTAAAAGTGG |  |  |
|  |  |  |  |  |
| **Chloramphenicol/florfenicol ARGs** | | | | |
| *catI* | FCATI | CT ATA ACC AGA CCG TTC AGC T | 499 | This work |
|  | RCATI | TAA GCA TTC TGC CGA CAT GGA |  |  |
| *floR* | FFLO | GTT TCA GGT GGC ACG AAA CC | 417 | This work |
|  | RFLO | CGG ACA CCG TGA AGA CAA TA |  |  |
| **Quinolones ARGs** |  |  |  |  |
| *qnrA* | FqnrA | ATTTCTCACGCCAGGATTTG | 516 | (Gay et al., 2006) |
|  | RqnrA | GATCGGCAAAGGTTAGGTCA |  |  |
| *qnrB* | FqnrB | GATCGTGAAAGCCAGAAAGG | 469 | (Gay et al., 2006) |
|  | RqnrB | ACGATGCCTGGTAGTTGTCC |  |  |
| *qnrS* | FqnrS | ACGACATTCGTCAACTGCAA | 417 | (Gay et al., 2006) |
|  | RqnrS | TAAATTGGCACCCTGTAGGC |  |  |
| *qnrD* | FqnrD | CGAGATCAATTTACGGGGAAT | 582 | (Xia et al., 2010) |
|  | RqnrD | AACAAGCTGAAGCGCCTG |  |  |
| *qepA1* | FqepA1 | GCAGGTCCAGCAGCGGGTAG | 218 | (Yamane et al., 2008) |
|  | RqepA1 | CTTCCTGCCCGAGTATCGTG |  |  |
|  |  |  |  |  |
| **Glycopeptides ARGs** |  |  |  |  |
| *vanA* | FvanA | TCAGCTTTGCATGGCAAGTC | 520 | (Dutka-Malen et al., 1995) |
|  | RvanA | GCTCCTCTGCTGAAAGGTCT |  |  |
| *vanB* | FvanB | CGGCAGGACAATATGATGGA | 419 | (Dutka-Malen et al., 1995) |
|  | RvanB | GCTGTCAATCAGTGCAGGAA |  |  |
| *vanC1* | FvanC1 | GGTATCAAGGAAACCTC | 822 | (Dutka-Malen et al., 1995) |
|  | RvanC1 | CTTCCGCCATCATAGCT |  |  |
| *vanC2/3* | FvanC23 | CTCCTACGATTCTCTTG | 439 | (Dutka-Malen et al., 1995) |
|  | RvanC23 | CGAGCAAGACCTTTAAG |  |  |
| *vanD* | FvanD | CATCAGGAAGCACAGCC | 235 | (Domingo et al., 2005) |
|  | RvanD | GCTGCTGTCATCATGCG |  |  |
| *vanE* | FvanE | TGTAGGTTGTGGTATCGGA | 515 | (Domingo et al., 2005) |
|  | RvanE | ATTCTCGCTAATCCTTTGCA |  |  |
| *vanG* | FvanG | GATGAAATCGAACTGTCAAG | 270 | (Domingo et al., 2005) |
|  | RvanG | AATGCCTTTCATCATATTTGG |  |  |
|  |  |  |  |  |
| **Heavy metal resistance genes** | | | | |
| **Arsenic** |  |  |  |  |
| *arsB* | arsB_F | GTSAARCCSTTYTCGATGGC | 226 | (Roosa et al., 2014) |
|  | arsB_R | GCRAASGCSAHSAYCATGAT |  |  |
|  |  |  |  |  |
| *arsC* | arsC_F | GTAATACGCTGGAGATGATCCG | 409 | (Sultan et al., 2020) |
|  | arsC_R | TTTTCCTGCTTCATCAACGAC |  |  |
|  |  |  |  |  |
|  |  |  |  |  |
| *arrA* | arrA_F | AAGGTGTATGGAATAAAGCGT TTG TBGGHGAYTT | 160-200 | (Escudero et al., 2013) |
|  | arrA_R | CCTGTGATTTCAGGTGCCCAYTYV GGNGT |  |  |
|  |  |  |  |  |
| **Copper** |  |  |  |  |
| *copA* | copA_F | ATGTGGAACSARATGCGKATGA | 193 | (Roosa et al., 2014) |
|  | copA_R | AGYTTCAGGCCSGGAATACG |  |  |
|  |  |  |  |  |
| **Nickel/cobalt/cadmium** |  |  |  |  |
| *nccA* | nccA_F | TTYAGCCAGGTVACSGTSATYTT | 532 | (Roosa et al., 2014) |
|  | nccA_R | GCYGCRTCSGCRCGCACCAGRTA |  |  |
|  |  |  |  |  |
| **Lead** |  |  |  |  |
| *pbrT* | pbrT_F | AGCGCGCCCAGGAGCGCAGCG TCTT | 448 | (Roosa et al., 2014) |
|  | pbrt_R | GGC TCG AAG CCG TCG AGRTA |  |  |
|  |  |  |  |  |
| **Chromium** |  |  |  |  |
| *chrB* | chrB_F | GTCGTTAGCTTGCCAACATC | 450 | (Adekanmbi et al., 2019) |
|  | chrB_R | CGGAAAGCAAGATGTCGATCG |  |  |
|  |  |  |  |  |
| **Integrases genes** |  |  |  |  |
| *IntI1* | FIntI1 | GGGTCAAGGATCTGGATTTCG | 484 | (Marathe et al., 2013) |
|  | RIntI1 | ACATGCGTGTAAATCATCGTC |  |  |
| *IntI2* | FIntI2 | TTACCTGCACTGGATTAAGC | 288 | (Abbasi et al., 2020) |
|  | RIntI2 | TTGCGAGTATCCATAACCTG |  |  |

| 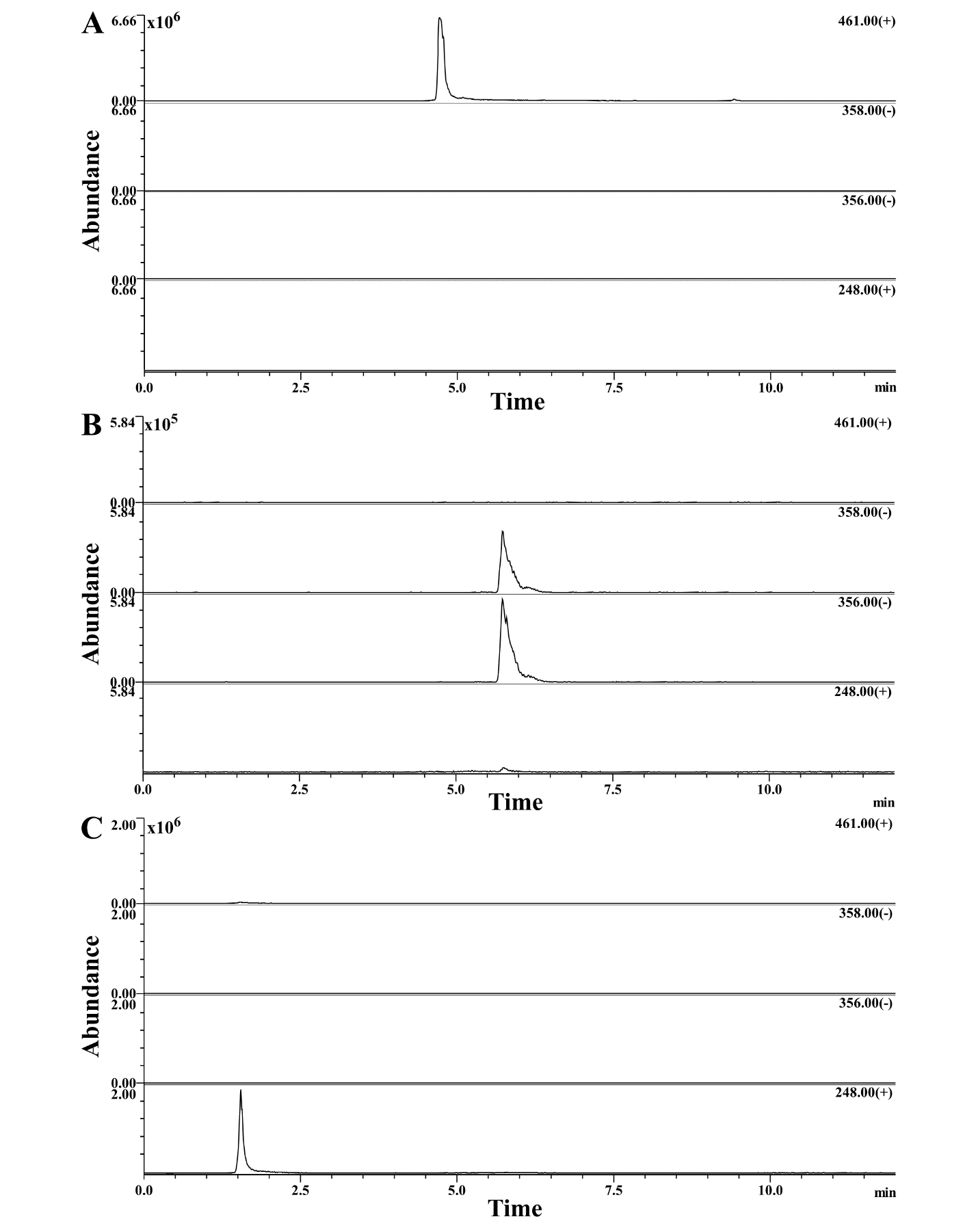  **Figure S1**. HPLC-MS analysis of the oxytetracycline (A), florfenicol (B), and florfenicol amine (C) standards. EIC of m/z 461 [M+H]+ corresponds to oxytetracycline, EIC of m/z 358 [M-H]- and 356 [M-H]- correspond to florfenicol, and EIC of m/z 248 [M+H]+ corresponds to florfenicol amine. |
| --- |

| 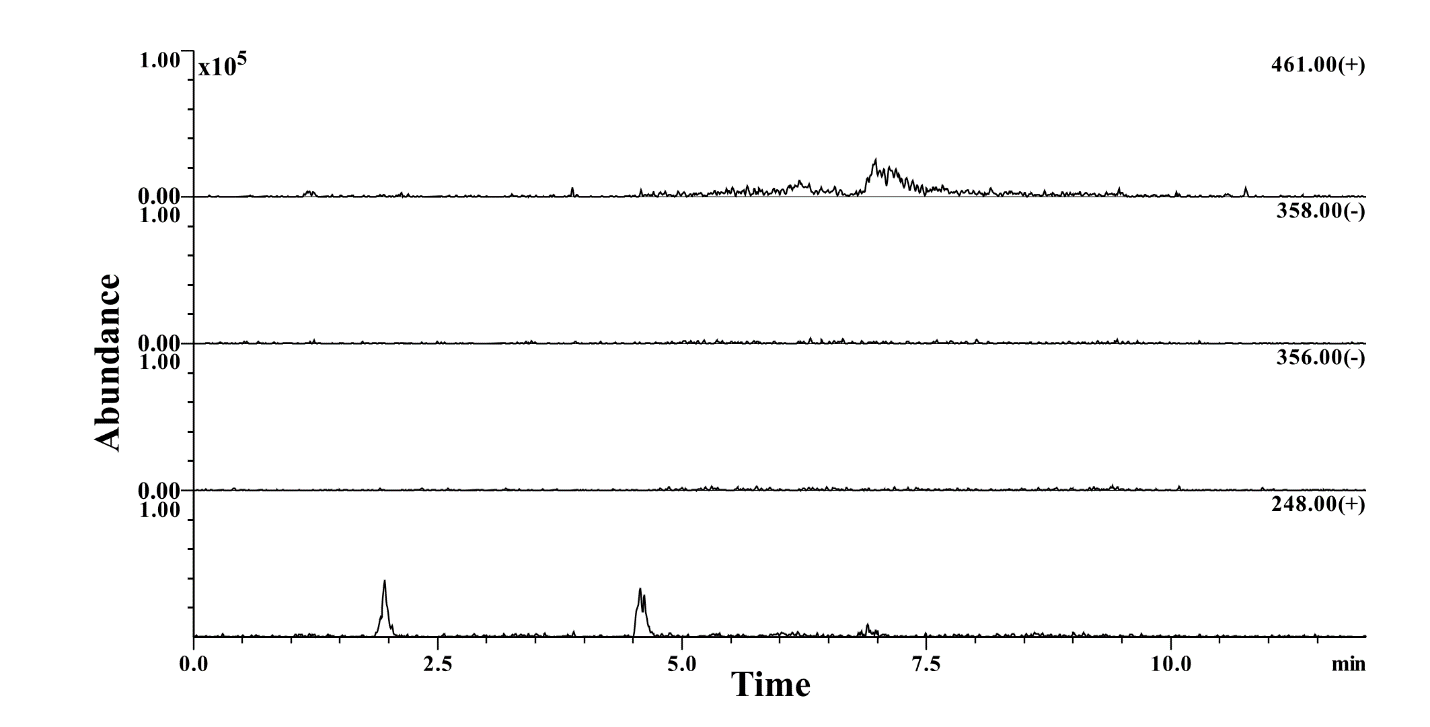 |
| --- |
| **Figure S2.** HPLC-MS analysis of the sediment sample no. B5. EIC of m/z 461 [M+H]+ corresponds to oxytetracycline, EIC of m/z 358 [M-H]- and 356 [M-H]- correspond to florfenicol, and EIC of m/z 248 [M+H]+ corresponds to florfenicol amine. |

| 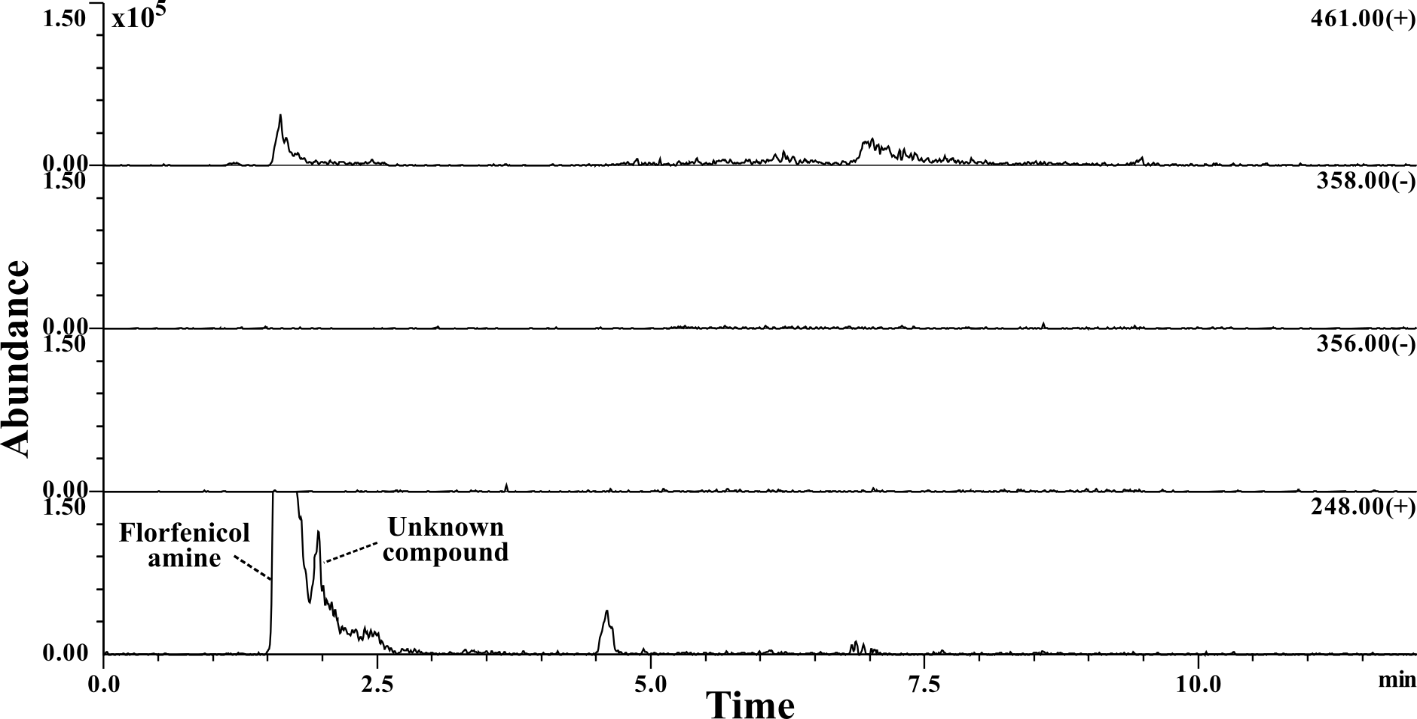 |
| --- |
| **Figure S3**. HPLC-MS analysis of the sediment sample no. B5 spiked with florfenicol amine. EIC of m/z 461 [M+H]+ corresponds to oxytetracycline, EIC of m/z 358 [M-H]- and 356 [M-H]- correspond to florfenicol, and EIC of m/z 248 [M+H]+ corresponds to florfenicol amine. |

## References

Abbasi, E., mondanizadeh, M., van Belkum, A., and Ghaznavi-Rad, E. (2020). Multi-Drug-Resistant Diarrheagenic Escherichia coli Pathotypes in Pediatric Patients with Gastroenteritis from Central Iran. *Infect. Drug Resist.* Volume 13, 1387–1396. doi:10.2147/IDR.S247732.

Adekanmbi, A. O., Adelowo, O. O., Okoh, A. I., and Fagade, O. E. (2019). Metal-resistance encoding gene-fingerprints in some bacteria isolated from wastewaters of selected printeries in Ibadan, South-western Nigeria. *J. Taibah Univ. Sci.* 13, 266–273. doi:10.1080/16583655.2018.1561968.

Armalytė, J., Skerniškytė, J., Bakienė, E., Krasauskas, R., Šiugždinienė, R., Kareivienė, V., et al. (2019). Microbial Diversity and Antimicrobial Resistance Profile in Microbiota From Soils of Conventional and Organic Farming Systems. *Front. Microbiol.* 10, 892. doi:10.3389/fmicb.2019.00892.

Chen, S., Zhao, S., McDermott, P. F., Schroeder, C. M., White, D. G., and Meng, J. (2005). A DNA microarray for identification of virulence and antimicrobial resistance genes in Salmonella serovars and Escherichia coli. *Mol. Cell. Probes* 19, 195–201. doi:10.1016/j.mcp.2004.11.008.

Chen, S., Zhao, S., White, D. G., Schroeder, C. M., Lu, R., Yang, H., et al. (2004). Characterization of Multiple-Antimicrobial-Resistant Salmonella Serovars Isolated from Retail Meats. *Appl. Environ. Microbiol.* 70, 1–7. doi:10.1128/AEM.70.1.1-7.2004.

Domingo, M.-C., Huletsky, A., Giroux, R., Boissinot, K., Picard, F. J., Lebel, P., et al. (2005). High prevalence of glycopeptide resistance genes vanB, vanD, and vanG not associated with enterococci in human fecal flora. *Antimicrob. Agents Chemother.* 49, 4784–4786. doi:10.1128/AAC.49.11.4784-4786.2005.

Dutka-Malen, S., Evers, S., and Courvalin, P. (1995). Detection of glycopeptide resistance genotypes and identification to the species level of clinically relevant enterococci by PCR. *J. Clin. Microbiol.* 33, 1434.

Eckert, C., Gautier, V., and Arlet, G. (2006). DNA sequence analysis of the genetic environment of various blaCTX-M genes. *J. Antimicrob. Chemother.* 57, 14–23. doi:10.1093/jac/dki398.

Escudero, L. V., Casamayor, E. O., Chong, G., Pedrós-Alió, C., and Demergasso, C. (2013). Distribution of Microbial Arsenic Reduction, Oxidation and Extrusion Genes along a Wide Range of Environmental Arsenic Concentrations. *PLoS ONE* 8, e78890. doi:10.1371/journal.pone.0078890.

Fang, H., Ataker, F., Hedin, G., and Dornbusch, K. (2008). Molecular Epidemiology of Extended-Spectrum β-Lactamases among Escherichia coli Isolates Collected in a Swedish Hospital and Its Associated Health Care Facilities from 2001 to 2006. *J. Clin. Microbiol.* 46, 707–712. doi:10.1128/JCM.01943-07.

Galimand, M., Lambert, T., Gerbaud, G., and Courvalin, P. (1993). Characterization of the aac(6’)-Ib gene encoding an aminoglycoside 6’-N-acetyltransferase in Pseudomonas aeruginosa BM2656. *Antimicrob. Agents Chemother.* 37, 1456–1462.

Gay, K., Robicsek, A., Strahilevitz, J., Park, C. H., Jacoby, G., Barrett, T. J., et al. (2006). Plasmid-mediated quinolone resistance in non-Typhi serotypes of Salmonella enterica. *Clin. Infect. Dis. Off. Publ. Infect. Dis. Soc. Am.* 43, 297–304. doi:10.1086/505397.

Machado, E., Coque, T. M., Cantón, R., Baquero, F., Sousa, J. C., Peixe, L., et al. (2006). Dissemination in Portugal of CTX-M-15-, OXA-1-, and TEM-1-producing Enterobacteriaceae strains containing the aac(6’)-Ib-cr gene, which encodes an aminoglycoside- and fluoroquinolone-modifying enzyme. *Antimicrob. Agents Chemother.* 50, 3220–3221. doi:10.1128/AAC.00473-06.

Madsen, L., Aarestrup, F. M., and Olsen, J. E. (2000). Characterisation of streptomycin resistance determinants in Danish isolates of Salmonella Typhimurium. *Vet. Microbiol.* 75, 73–82. doi:10.1016/S0378-1135(00)00207-8.

Marathe, N. P., Regina, V. R., Walujkar, S. A., Charan, S. S., Moore, E. R. B., Larsson, D. G. J., et al. (2013). A Treatment Plant Receiving Waste Water from Multiple Bulk Drug Manufacturers Is a Reservoir for Highly Multi-Drug Resistant Integron-Bearing Bacteria. *PLoS ONE* 8, e77310. doi:10.1371/journal.pone.0077310.

Maynard, C., Fairbrother, J. M., Bekal, S., Sanschagrin, F., Levesque, R. C., Brousseau, R., et al. (2003). Antimicrobial resistance genes in enterotoxigenic Escherichia coli O149:K91 isolates obtained over a 23-year period from pigs. *Antimicrob. Agents Chemother.* 47, 3214–3221.

Ng, L. K., Martin, I., Alfa, M., and Mulvey, M. (2001). Multiplex PCR for the detection of tetracycline resistant genes. *Mol. Cell. Probes* 15, 209–215. doi:10.1006/mcpr.2001.0363.

Ramirez, M. S., and Tolmasky, M. E. (2010). Aminoglycoside modifying enzymes. *Drug Resist. Updat. Rev. Comment. Antimicrob. Anticancer Chemother.* 13, 151–171. doi:10.1016/j.drup.2010.08.003.

Roosa, S., Wattiez, R., Prygiel, E., Lesven, L., Billon, G., and Gillan, D. C. (2014). Bacterial metal resistance genes and metal bioavailability in contaminated sediments. *Environ. Pollut.* 189, 143–151. doi:10.1016/j.envpol.2014.02.031.

Seputiene, V., Bogdaite, A., Ruzauskas, M., and Suziedeliene, E. (2012). Antibiotic resistance genes and virulence factors in Enterococcus faecium and Enterococcus faecalis from diseased farm animals: pigs, cattle and poultry. *Pol. J. Vet. Sci.* 15, 431–438.

Šeputiene, V., Ružauskas, M., Žlabys, P., and Sužiedėlienė, E. (2006). Characterisation of streptomycin resistance determinants in Lithuanian Escherichia coli isolates. *Biologija* 2, 14–17.

Sultan, I., Ali, A., Gogry, F. A., Rather, I. A., Sabir, J. S. M., and Haq, Q. M. R. (2020). Bacterial isolates harboring antibiotics and heavy-metal resistance genes co-existing with mobile genetic elements in natural aquatic water bodies. *Saudi J. Biol. Sci.* 27, 2660–2668. doi:10.1016/j.sjbs.2020.06.002.

Vakulenko, S. B., Donabedian, S. M., Voskresenskiy, A. M., Zervos, M. J., Lerner, S. A., and Chow, J. W. (2003). Multiplex PCR for detection of aminoglycoside resistance genes in enterococci. *Antimicrob. Agents Chemother.* 47, 1423–1426.

Xia, L.-N., Li, L., Wu, C.-M., Liu, Y.-Q., Tao, X.-Q., Dai, L., et al. (2010). A survey of plasmid-mediated fluoroquinolone resistance genes from Escherichia coli isolates and their dissemination in Shandong, China. *Foodborne Pathog. Dis.* 7, 207–215. doi:10.1089/fpd.2009.0378.

Yamane, K., Wachino, J., Suzuki, S., and Arakawa, Y. (2008). Plasmid-mediated qepA gene among Escherichia coli clinical isolates from Japan. *Antimicrob. Agents Chemother.* 52, 1564–1566. doi:10.1128/AAC.01137-07.
